# Testing a breast cancer prevention and a multiple disease prevention weight loss programme amongst women within the UK NHS breast screening programme—a randomised feasibility study

**DOI:** 10.1186/s40814-021-00947-4

**Published:** 2021-12-20

**Authors:** Michelle Harvie, David P. French, Mary Pegington, Grace Cooper, Anthony Howell, Sarah McDiarmid, Cheryl Lombardelli, Louise Donnelly, Helen Ruane, Katharine Sellers, Emma Barrett, Christopher J. Armitage, D. Gareth Evans

**Affiliations:** 1 The Prevent Breast Cancer Research Unit, The Nightingale Centre, Manchester University NHS Foundation Trust, Manchester, M23 9LT UK; 2 Present Address: Manchester Breast Centre, Oglesby Cancer Research Centre, The Christie, University of Manchester, 555 Wilmslow Rd., Manchester, M20 4GJ UK; 3 Manchester Centre for Health Psychology, School of Health Sciences, University of Manchester, Coupland Street, Manchester, M13 9PL UK; 4 Division of Cancer Sciences, The University of Manchester, Wilmslow Road, Manchester, M20 4BX UK; 5 Department of Medical Oncology, The Christie NHS Foundation Trust, Wilmslow Rd., Manchester, M20 4BX UK; 6 Department of Medical Statistics, Education and Research Centre, Manchester University NHS Foundation Trust, M23 9LT, Manchester, UK; 7 Genomic Medicine, Division of Evolution and Genomic Sciences, The University of Manchester, St Mary’s Hospital, Manchester University NHS Foundation Trust, Oxford Road, Manchester, M13 9WL UK

**Keywords:** Breast screening, Breast cancer, Weight loss, Behaviour change, Cardiovascular risk, NHS Health Check, diabetes risk, Website and phone programme

## Abstract

**Background:**

Excess weight and unhealthy behaviours (e.g. sedentariness, high alcohol) are common amongst women including those attending breast screening. These factors increase the risk of breast cancer and other diseases. We tested the feasibility and acceptability of a weight loss/behaviour change programme framed to reduce breast cancer risk (breast cancer prevention programme, BCPP) compared to one framed to reduce risk of breast cancer, cardiovascular disease (CVD) and diabetes (T2D) (multiple disease prevention programme, MDPP).

**Methods:**

Women aged 47-73 years with overweight or obesity (*n* = 1356) in the NHS Breast Screening Programme (NHSBSP) were randomised (1:2) to be invited to join a BCPP or a MDPP. The BCPP included personalised information on breast cancer risk and a web and phone weight loss/behaviour change intervention. The MDPP also included an NHS Health Check (lipids, blood pressure, HbA1c and personalised feedback for risk of CVD [QRISK2] and T2D [QDiabetes and HbA1c]). Primary outcomes were uptake and retention and other feasibility outcomes which include intervention fidelity and prevalence of high CVD and T2D risk. Secondary outcomes included change in weight.

**Results:**

The BCPP and MDPP had comparable rates of uptake: 45/508 (9%) vs. 81/848 (10%) and 12-month retention; 33/45 (73%) vs. 53/81 (65%). Both programmes had a high fidelity of delivery with receipt of mean (95% CI) 90 (88-98% of scheduled calls, 91 (86-95%) of scheduled e-mails and 89 (76-102) website entries per woman over the 12-month period. The MDPP identified 15% of women with a previously unknown 10-year CVD QRISK2 of ≥ 10% and 56% with 10-year Qdiabetes risk of ≥ 10%. Both groups experienced good comparable weight loss: BCPP 26/45 (58%) and MDPP 46/81 (57%) with greater than 5% weight loss at 12 months using baseline observation carried forward imputation.

**Conclusions:**

Both programmes appeared feasible. The MDPP identified previously unknown CVD and T2D risk factors but does not appear to increase engagement with behaviour change beyond a standard BCPP amongst women attending breast screening. A future definitive effectiveness trial of BCPP is supported by acceptable uptake and retention, and good weight loss.

**Trial registration:**

ISRCTN91372184, registered 28 September 2014.

**Supplementary Information:**

The online version contains supplementary material available at 10.1186/s40814-021-00947-4.

## Uncertainties regarding feasibility that existed prior to this study

Can we recruit and engage breast screening attendees to a web and phone weight loss/behaviour change breast cancer or multiple disease prevention programmes?

What proportion of women have previously unidentified high risks of CVD and T2D?

The fidelity of delivery and patient satisfaction with the programmes

Are there any associated harms of the programmes, e.g. increased anxiety?

Which of the programmes should be tested further in a definitive large scale RCT within the NHSBSP?

## Key feasibility findings from this study

The MDPP and BCPP web/phone programmes appeared feasible.

The MDPP identified previously unknown CVD and T2D risk factors but does not appear to increase engagement with behaviour change beyond a standard BCPP

No evidence of harms.

## Implications of the findings on the design of the main study

A future definitive effectiveness trial of BCPP is supported by acceptable uptake and retention, and good weight loss.

## Background

Women in the UK aged between 50 and 70 years are invited for breast screening as part of the National Health Service Breast Screening Programme (NHSBSP). In common with women of this age in the general population, many attendees have overweight or obesity (62%) [1], sedentariness (80%) [1], high alcohol intakes (11.5%) [1], sub-optimal diets (80%) [2] and smoke (16%) [3]. It is estimated that these factors combined may be responsible for 30% of breast cancers [4] and significant proportions of other multifactorial conditions including 70% of CVD [5], and 90% of T2D [6]. Unhealthy behaviours and the associated disease risks are not currently addressed within the NHSBSP.

There are currently moves to introduce breast cancer (BC) risk assessment to the NHSBSP in order to identify higher risk women for chemoprevention and risk adapted breast screening [7–10]. The Predicting Risk of Cancer at Screening (PROCAS) study reported up to 21% of 57,902 in the Greater Manchester NHSBSP are at either high or above average risk of BC (10-year risk of ≥ 5%), 50% are at average risk (10-year risk of 2-4.9%) and 29% are at low risk (10-year risk < 2%) [11].

Women of breast screening age in the UK are also eligible for assessment of CVD and T2D risk assessment as part of the NHS Health Check. Current coverage and outcomes for this programme are sub-optimal as it is mainly opportunistic [12]. Public Health England estimates that only 49% of adults with raised cholesterol and 57% with hypertension have been identified. Furthermore, low proportions of those with raised levels currently receive appropriate statin (35%) or anti-hypertensive (49%) medications [13]. The programme is currently being reviewed with an aim to improve delivery and outcomes of the checks [14]. Offering CVD and T2D risk assessment and a behaviour change programme at breast screening could provide systematic access to CVD and T2D care pathways due to high NHSBSP coverage and thus potentially better outcomes.

The PROCAS Lifestyle study reported here aimed to explore the feasibility of BCPP and MDPP for women with overweight/obesity following receipt of BC risk estimates as part of risk stratified screening. A sample of women with overweight/obesity participating in the PROCAS study were randomised to receive an invitation to join a standard web and phone Breast Cancer Prevention Programme (BCPP) which reminded women of their personal risk of BC or a Multiple Disease Prevention Programme (MDPP) which additionally included an NHS Health Check to assess lipids, blood pressure and Hba1c; provided personalised risk feedback for CVD, T2D and emphasised the links between health behaviours and dementia. The programmes asked women to follow either a daily or intermittent (5:2) energy restricted Mediterranean diet. Both approaches have found to be effective in our previous weight loss studies amongst high risk women [15], and are equally effective for long-term weight loss success in a variety of populations [16].

We previously reported that higher BC risk predicted uptake retention and weight loss success across both BCPP and MDPP [17]. We now report on the main trial aim, which is to assess the feasibility of the intervention and trial methods. The primary objectives were uptake and retention. Other feasibility objectives included predictors of retention, completeness of data collection, the need for the programmes (the number of participants who have previously had an NHS Health Check, numbers at high risk of CVD and T2D and evidence of uptake equity across demographic groups), fidelity of intervention delivery, participant satisfaction and any associated harms of the programmes, e.g. anxiety and health status. We also report preliminary efficacy data for change in weight, which is the planned primary outcome for a subsequent definitive trial, and other lifestyle behaviours.

A final objective was to determine which of these programmes should be tested further in a definitive large scale RCT within the NHSBSP.

## Method

### Study design

We conducted a single centre prospective two arm randomised trial (1:2) of BCPP vs. MDPP amongst women attending the NHSBSP, and who had participated in the PROCAS study. Recruitment was between November 2014 and October 2015. This period was a median (range) 55 (5-72) months after completing BC risk assessments and 6.5 (0-63) months after receiving personalised BC risk feedback as part of the PROCAS study. The long lag between risk assessment and feedback was because of the need to validate the Tyrer-Cuzick risk assessment model amongst women attending the NHSBSP. All trial procedures were undertaken at the Prevent Breast Cancer Research Unit at Wythenshawe Hospital, Manchester University NHS Foundation Trust.

### Patient and public involvement

The trial design and participant resources and pathway were designed in collaboration with a panel of women attending breast screening. Four members of this panel were part of the trial management group.

### Recruitment and randomisation

**Fig. 1:**
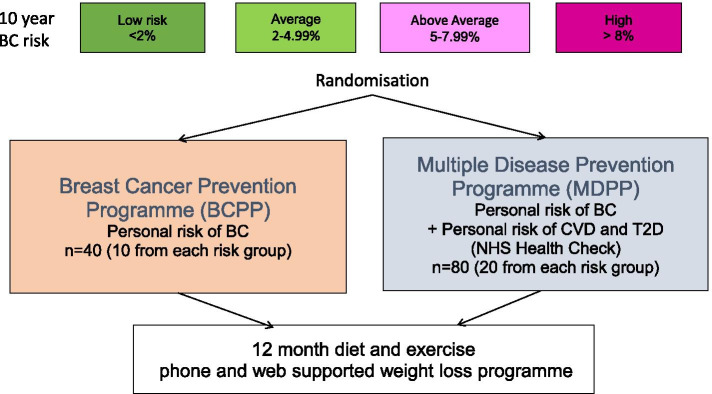
Study design

The study included women with overweight/obesity (BMI ≥ 25 kg/m^2^) aged 47–73 years who had been identified and informed they were at high (10-year risk ≥ 8%), above average (5-7.9%), average (2-4.9%) or low-risk (< 2%) of BC as described previously [1]. The personalised estimate of their risk of BC had been derived using the Tyrer-Cuzick model (version-8 which includes family history, hormonal risk factors, BMI and visually assessed mammographic density [11]). Women were randomised 1:2 to receive a mailed invitation to either the BCPP or MDPP programmes. We included a larger MDPP group to assess any issues that may arise when participants are given this novel programme. Randomisation to the two different invitations allowed us to assess uptake separately to the two programmes (Fig. 1). Interested women were asked to check their eligibility on the trial website, or to ring the trial office if they did not have immediate access to the internet. Women were excluded if they did not have access to a phone or the internet; had a previous diagnosis of cancer, T2D or CVD; were currently prescribed statins; had a major physical or psychiatric condition which made them unsuitable for a home based diet and physical activity (PA) programme or were current users of HRT since weight only affects risk of BC amongst non-HRT users [18]. The invitation letter included an opt-out slip to indicate reasons they were not eligible or not interested.

The sample size of 40 in the BCPP group allowed us to estimate an uptake of 10% (CI +/−9.5%) and a retention rate of 60% (CI +/−15.0%). We recruited double the number of participants in the novel MDPP group (*n* = 80). The final sample size of 120 (40 BCPP and 80 MDPP) included 10 women from each BC risk group to the BCPP and 20 from each BC risk group to the MDPP.

Participant randomisation was undertaken independently from the research team by an independent member of the Department of Statistics at Manchester University NHS Foundation Trust to ensure allocation concealment using nQuery Advisor 7.0. The first batch used a 1:2 randomisation of BCPP to MDPP across the four BC risk groups. Subsequent randomisations were adaptive to the response rate of each risk category in the two groups until 45 women were randomised to the BCPP group and 81 to the MDPP group.

### The BCPP and MDPP programmes

The programmes are described in Table 1. Both programmes were delivered by research dietitians who had undergone training on communicating personalised risk of BC, CVD, T2D and dementia and the lifestyle prevention of these conditions. Both programmes reminded women of their personal BC risk. In addition, the MDPP provided personalised risk of developing CVD (10-year and lifetime risk and heart age from QRISK2 [19]) and T2D (10 year risk from QDiabetes [20] and measured HbA1c).

**Table 1:** The BCPP and MDPP programmes

Timeline		Health care professional	Modality of contact	BCPP	MDPP
	**Disease risk information**				
Median (range)-6.5 (0 – 63) months	Personalised breast cancer risk information had been received prior to invitation to the lifestyle programmes (10 year and lifetime – derived from the Tyrer-Cuzick model)	Clinician in the high risk clinic (High or above average risk) or Letter (average or below average risk)	Face to face / phone Letter	✓	✓
Time 0	Reminder of personalised breast cancer risk	Research dietitian	Face to face	✓	✓
	Advice that weight loss of ≥5% and adherence to PA and alcohol recommendations could lead to significant reductions in risk of BC (25%) [21, 22]	Research dietitian	Face to face	✓	✓
	General advice that 5% weight loss could reduce their risk of T2D (60%) [23] and CVD (30%) [24]	Research dietitian	Face to face	✓	
	NHS health check tests	Research dietitian/research practitioner	Face to face		✓
	Personalised feedback of: CVD risk (10-year and lifetime risk and heart age from QRISK2) [19] T2D risk (QDiabetes and measured HbA1c) [20]	Research dietitian	Face to face		✓
	Personalised estimate of change in CVD risk from predicted reductions in blood pressure and total cholesterol) (i.e. a 1 mm/Hg reduction in systolic blood pressure per 1% weight loss up to a 10% weight loss [25] and a 1% reduction in total cholesterol for every 1% weight loss up to a 15% weight loss) [26]. Personalised estimate of change in Q diabetes risk by entering the target reduced weight in the QDiabetes tool	Research dietitian	Face to face		✓
	NHS Health Check results sent to general practitioner to allow appropriate follow up and clinical management, e.g. checking abnormal result, consideration of medications for raised cholesterol, blood pressure and HbA1c.	General practitioner	Face to face/phone		✓
	**Lifestyle advice and behavioural support **				
Time 0	Dietary advice to follow an intermittent (5:2) or daily energy restricted Mediterranean diet	Research dietitian	Face to face	✓	✓
	Physical activity advice (150 mins/moderate intensity CV & 40 mins of resistance exercise /week) [27] tailored to participant’s preferences, abilities and co-morbidities, with referral to local services where appropriate^a^	Research dietitian	Face to face	✓	✓
	Advised to limit alcohol intake to <10 units/week due to its effect on weight and weight independent effects on disease risk		Face to face	✓	✓
	Tutorial for use of the trial website & self-monitoring	Research dietitian	Face to face	✓	✓
	Referral to NHS smoking cessation or alcohol services if reporting high-risk alcohol intakes with Alcohol Use Disorders Identification Test (AUDIT) scores >8 [28]	Participants asked to self- refer to relevant services	Face to face	✓	✓
0 – 6 months	Scheduled review calls at weeks 1, 4 and 8	Research dietitian	Phone	✓	✓
	Weekly personalised e mails (weeks 2,3,5,6,7,9-26)	Research dietitian	E mail	✓	✓
	Monthly trial newsletter	Automated	E mail	✓	✓
	3 and 6 month weight review	Research dietitian	Face to face	✓	✓
	Self-management using the trial website	Participants	Web	✓	✓
6- 12 months	Automated monthly email from the trial website: Positive feedback for records showing weight loss or weight maintenance Encouraged re-engagement with the programmes if weight had increased by ≥1kg or if no website entries recorded.	Automated	E mail	✓	✓
	Self -management using the trial website	Participants	Web	✓	✓

^a^Suitability to follow a home based PA programme was confirmed using the adult pre-physical activity screening system tool [29], with family doctor clearance where necessary

Both programmes promoted weight loss of ≥ 5% and communicated how weight loss and health behaviour change could reduce risk of breast cancer [21, 22], type 2 diabetes [23] and CVD [24]. This was presented as a gain-framed message to increase response efficacy [30].

The programmes were supported by a trial website which encouraged women to self-monitor and record their weight, diet (completion of restricted days in the 5:2 diet and actual food and drink intake) and PA (both cardiovascular and resistance). It hosted weekly menu plans, recipes, information about BC, CVD, T2D and dementia, tips for planning and managing emotional eating, online videos of the recommended exercises (Physiotec, Canada) and a monthly newsletter to maintain engagement. Women received tailored feedback on their self-reported behaviours from their allocated trial dietitian in scheduled phone calls (weeks 1, 4, 8) and weekly personalised e-mails for 6 months. They were invited to enter an optional continued weight loss/weight maintenance period between 6- and 12-month women when they received automated monthly e-mails in response to website entries.

Communicating personalised risk information without increasing self-efficacy or encouraging self-regulation is not likely to produce changes in behaviour [31]. Given this, both programmes included key behaviour change techniques as recommended in the UK NICE behaviour change guidance, including goal setting, planning, relapse prevention, self-monitoring and encouraging individuals to identify sources of social support for changing behaviours [32].

### Primary outcome measures

#### Uptake and retention

For each programme, we recorded the number of women who were invited and consented to join the study, and who were not interested or not eligible to take part and the reasons for this. Also, retention to the programmes at 3, 6 and 12 months.

#### Predictors of retention

Within the MDPP, we assessed whether being identified at different levels of CVD and T2D disease risk was associated with disengagement from behaviour change, evidenced by differences in withdrawal.

#### Completeness of weight and self-reported measures

We recorded the number of women with weight and body fat measures and who had completed self-report measures of physical activity (IPAQ) [33], alcohol intake and harms (AUDIT) [28], diet (7-day food diary), smoking status, health status (EQ-5D-5L) [34] and perceived self-rated anxiety (state trait anxiety) [35] during the study.

#### Need for the programmes

We assessed the prevalence of previously unknown raised CVD and T2D risk identified in the MDPP programme. Also, demographics of women recruited to the programmes including ethnicity and socioeconomic status using index of multiple deprivation (IMD) quintiles [36].

#### Fidelity of delivery and engagement to the programmes

We assessed the numbers of scheduled calls and e-mails received, engagement with the web site and the amount of dietitian time that was used to deliver the programmes. Also, the numbers of women in both groups referred to NHS behaviour change services (i.e. exercise on referral, alcohol and smoking cessation services) or commenced on statins or blood pressure medications.

#### Participant satisfaction

We collated anonymised 12-month participant questionnaires which rated satisfaction with the overall programme, study visits and the trial website. This included free text suggestions for how the programmes could be improved. A specific website feedback questionnaire was completed at 3 and 6 months to include feedback from women who might leave the programme before 12 months.

#### Potential harms of the programmes

Potential harms of both programmes were assessed in terms of serious or unexpected adverse events and changes in perceived self-rated anxiety (state trait anxiety) [35] and health status (EQ-5D-5L) [34].

### Secondary outcomes

#### Weight loss and health-related behaviour change

We assessed change in weight and body fat (Tanita MC-180MA, Tanita Europe, Amsterdam, The Netherlands) and the number and percentage of women losing 5% of weight or greater at 12 months. Also, self-reported PA (IPAQ) [33], alcohol harms and intake (AUDIT [28]), saturated fat intake (7-day food diary analysed using Wisp version 3 (Tinuviel Software, Anglesey, Wales) [37]) and smoking status in both groups at 3 and 6 months.

### Analysis

Descriptive statistics of uptake and retention, fidelity of the intervention, participant satisfaction and changes in weight and health behaviours in the MDPP and BCPP groups were undertaken. Multivariable logistic regression was used to assess the association between estimated level of CVD and T2D risk and the likelihood of withdrawal at 12 months within the MDPP group. This was assessed in terms of 10-year CVD QRISK2, heart age compared to actual age, having a first degree relative with CVD, 10-year QDiabetes and having a first degree relative with diabetes. A priori confounding variables included BC risk (high, above average, average and low), index of multiple deprivation (IMD quintiles; 1, 2 and 3-5) [36], smoking status (never, ex-smoker and current smoker), age and BMI at baseline.

Baseline observation carried forward (BOCF) values are reported for changes in weight (% and kg), body fat (kg), alcohol intake and PA. This provides an estimate of intention to treat assuming that people who leave an intervention are likely to revert to their baseline weight and behaviours. Completers only analysis was undertaken for state anxiety and self-rated health status.

Given the relatively large numbers in this feasibility trial, we undertook exploratory formal statistics to assess comparisons of changes in these parameters relative to baseline between the BCPP and MDPP conditions. The findings of these are reported as an indication of the effects of the two programmes with the caveat; there is multiple testing of secondary outcomes and the study is not necessarily powered to show differences.

*P* < 0.05 was considered statistically significant for all analyses. All statistical analyses were conducted using SPSS version 23 (IBM Corp., Armonk, NY, USA), R version 3.5.1 and Stata Statistical Software: Release 16.

## Results

### Recruitment and retention to the programmes

**Fig. 2:**
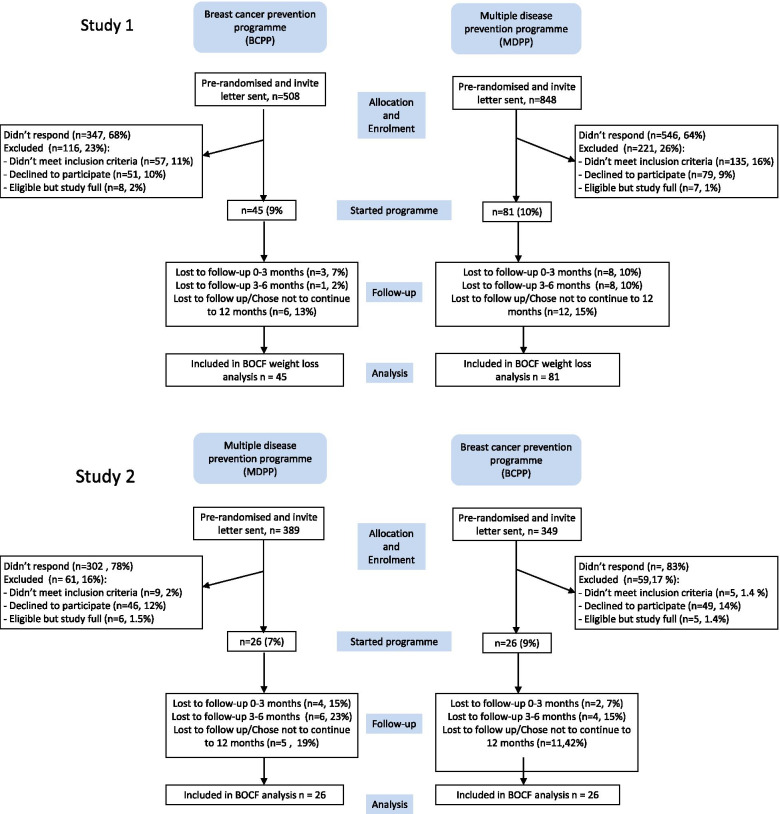
Consolidated Standards of Reporting Trials (CONSORT) flow diagram of participants recruited to the PROCAS lifestyle trial

Uptake to the study was comparable with invitations to both the BCPP and MDPP (45/508 9%; vs. 81/848 10%, *p* = 0.7) (Fig. 2). An additional 57 women invited to the BCPP (11%) and 135 invited to the MDPP (16%) were interested in joining but were not eligible. This was mainly due to existing health problems such as previous CVD, cancer and raised cholesterol (7.1% BCPP, 12.0% MDPP). Other reasons for declining included lack of internet access (1.4% BCPP, 0.8% MDPP) and current use of HRT (1.0% BCPP, 1.2% MDPP). A further 8 women invited to the BCPP (2%) and 7 invited to the MDPP (1%) wished to enter the study but responded after recruitment was complete.

An additional 130 women (10%) invited to both programmes returned a response slip to say they were not interested in taking part. The most common reasons cited were lack of time (28%), issues travelling to the centre for trial appointments (18%), family illness or caring duties (12%). Uptake to both programmes was greater in women at high and above average BC risk compared to average and low risk as reported previously (Table 2) [17].

**Table 2:** Uptake, retention and weight loss within the BCPP and MDPP groups across breast cancer risk categories

	BCPP					MDPP				
10-year risk of breast cancer	Invited, ***n***	Uptake, ***n*** (%)	Retention at 12 months, ***n*** (%)	Weight loss, %^**a**^	% Losing ≥ 5% weight, ***n*** (%)	Invited, ***n***	Uptake, ***n*** (%)	Retention at 12 months, ***n*** (%)	Weight loss, kg^**a**^	% Losing ≥ 5% weight, ***n*** (%)
**Low (< 2%)**	206	8 (3.9)	3 (37.5)	−4.2 (−9.7 to +1.3)	2 (25)	354	20 (5.6)	12 (60)	−6.8 (−10.6 to −3.0)	10 (50.0)
**Average (< 5 to > 2%)**	171	17 (9.9)	13 (76.5)	−7.0 (−11.1 to −3.0)	10 (58.8)	267	21 (7.9%)	16 (76)	−7.1 (−10.1 to −4.2)	12 (57.1)
**Above average (≥ 5-< 8%)**	59	10 (16.9)	9 (90.0)	−9.3 (−15.0 to −3.6)	6 (60)	127	20 (16)	11 (55)	−7.3 (−11.1 to −3.4)	11 (55.0)
**High (≥ 8%)**	72	10 (13.9)	8 (80.0)	−9.6 (−14.6 to −4.5)	8 (80)	100	20 (20)	14 (70)	−8.4 (−12.4 to −4.4)	13 (65.0)
**Total/average**	1356	45 (8.9)	33 (73.3)	−7.6 (−9.8 to −5.4)	26 (58)	738	81 (9.6)	53 (65.4)	−7.4 (−9.1 to −5.7)	46 (56.8)

^a^Mean (95% confidence interval) baseline carried forward percentage weight loss at 12 months

Five women in the BCPP (11%) and 16 women in the MDPP (20%) left the programme during the 6 months of phone and e-mail dietitian support (Fig. 2). A further 6 BCPP (14%) and 12 MDPP (15%) women left the programme during the automated e-mail support period between 6 and 12 months. Of the total women who left the programmes in the 12-month period, i.e. 11 (25%) in the BCPP and 28 (35%) in the MDPP groups, 7 BCPP (16%) and 20 MDPP (25%) asked to withdraw and a further 4 BCPP (9%) and 8 MDPP (10%) lost contact. The most commonly cited reasons for withdrawal included personal or family illness (*n* = 11, 43%), too busy (*n* = 5, 17%), and not able to adhere to the programme (*n* = 5, 17%) (Fig. 2).

### Predictors of retention to the BCPP and MDPP

Retention appeared to be greater amongst women at higher risk BC risk in the BCPP group, but was more variable across BC risk categories for the MDPP group (Table 2). Within the MDPP, women informed they had a higher CVD QRISK2 score at baseline were more likely to leave the programme. Adjusted odds ratio for leaving (95% CI) 1.36 (1.08, 1.80) for each unit increase in 10 year CVD QRISK2 score (*p* = 0.02) and 1.22 (1.03, 1.46) for each year of predicted heart age greater than their actual age (*p* = 0.02) (Supplementary Table 1).

### Completeness of weight and self-report measures

Weight measures were obtained in 100% of completers. There were high levels of completion for the self-report measures. The median response rate at 6 m for the self-report measures collected across both groups was 78% (Supplementary Table 2).

### Need for the programmes

Baseline characteristics of participants are reported in Table 3. The BCPP and MDPP groups were comparable. Women were mainly from the three least deprived quintiles of the index of multiple deprivation and white British. Approximately one-third of the cohort had a family history of BC (BCPP 26.7%, MDPP 34.6%,). Sixty-two percent of the BCPP and 60.5% MDPP groups had previously undertaken a commercial weight loss programme, and there were few smokers (BCPP 6.7% vs. MDPP 7.4%).

**Table 3:** Baseline characteristics of the BCPP and MDPP groups

	BCPP (*n* = 45)	MDPP (*n* = 81)
Age (years)^a^	59.1 (4.8) 51.7-73.7	60.3 (5.4) 51.2-72.0
BMI (kg/m^2^)^a^	31.1 (4.8) 25.6-46.9	31.6 (4.4) 25.0-43.8
Overweight^b^	23 (51.1)	37 (45.7)
Obese^b^	22 (48.9)	44 (54.3)
Townsend quintile^b^		
1 (least deprived)	12 (26.7)	28 (34.6)
2	21 (46.7)	31 (38.3)
3	9 (20)	16 (19.8)
4	3 (6.7)	4 (4.9)
5 (most deprived)	0 (0)	2 (2.5)
First degree relative with breast cancer^b^	12 (26.7)	28 (34.6)
Pre, peri/postmenopausal^b^	7 (15.6)/38 (84.4)	9 (11.1)/72 (88.9)
Current smoker^b^	3 (6.7)	6 (7.4)
Ethnicity^b^		
White British	42 (93.3)	80 (98.8)
Asian	2 (4.4)	0
Afro-Caribbean	1 (2.2)	1 (1.2)
Time since receiving breast cancer risk feedback (months)^a^	11.6 (15.6) 1-63	13.2 (13.7) 1-54
Previous commercial weight loss programme^b^	28 (62.2)	49 (60.5)
Previous primary care weight loss service^b^	2 (4.4)	4 (4.9)

^a^Mean (SD) minimum-maximum

^b^*N* (%)

Ten women (12.3%) in the MDPP group had previously had an NHS Health Check more than 6 months prior to joining the study. Relatively high numbers of women in the MDPP group had previously unknown increased risks for CVD (14.8% with ≥ 10% 10 year risks of CVD and 14.8% with total cholesterol ≥ 7.5 mmol/L) and for T2D (56% with ≥ 10% 10-year risk of T2D, and 6.2% with HbA1c > 42 mmol/mmol) (Table 4). Some of these women were subsequently commenced on medications for blood pressure (*n* = 2) and raised cholesterol (*n* = 1) by their family doctor.

**Table 4:** Previously unknown cardiovascular and diabetes risk markers and estimated risks at baseline in the MDPP

	Cardiovascular risk				Type-2 diabetes risk	
Breast cancer risk categories	10 year risk ≥ 10 %	Lifetime risk ≥ 25 %	Total cholesterol > 7.5 mmol/L	Systolic blood pressure > 140 mmHg	10 year risk ≥ 10%	HbA1c > 42 mmol/mmol
High/moderately increased (*n* = 40)	8 (20.0)	20 (50.0)	7 (17.5)	19 (47.5)	23 (57.5)	1 (2.5)
Average/below average (*n* = 41)	4 (10.0)	16 (39.0)	5 (12.2)	21 (51.2)	22 (53.7)	4 (9.8)
Total (*n* = 81)	12 (14.8)	36 (44.4)	12 (14.8)	40 (49.4)	45 (55.6)	5 (6.2)

*n* (%)

### Fidelity of programme delivery

Dietitian time for delivery of the intervention and fidelity of delivery are described in Table 5.

**Table 5:** Dietitian time and fidelity of delivery of the BCPP and MDPP programmes

Elements of the programmes	Numbers received/participants engaging, *n* (%)	
Initial personalised disease risk and diet and physical activity advice	126 (100%) BCPP 40 min dietetic time MDPP 60 min dietetic time	
Dietitian review calls week 1, 4 and 8—mean 95 (CI)% of calls received	90 (88-98%) 21 (6 to 50) min dietetic time	
Dietitian personalised e-mails 0-6 months—mean 95 (CI)% of e-mails sent	91 (86-95%) 15 (5 to 30) min dietetic time	
Web site use^a^		
Used baseline—3 months	122 (97%)	
Used 3-6 months	109 (87%)	
Used 6-9 months	105 (83%)	
Used 9-12 months	101 (79%)	
Received automated e-mail between 6 and 12 months for weight regain of > 1 kg	27% of women in the 6-12 month programme	
Number of website entries per participant across the programmes	89 (76-102)^a^	
Diet choice at start of the programme	5:2 125 (98%) Daily Mediterranean 1 (2%)	
Diet reported at 3 months	5:2 112 (89 %) Daily Mediterranean 1 (1%) No data 13 (10%)	
Diet reported at 6 months	5:2 67 (53%) Daily Mediterranean 1 (1%) No data 58 (46%)	
Referrals to NHS behaviour change services	BCPP	MDPP
Physical activity	8 (18%)	23 (28.4%)
Alcohol services	0	4 (4.9%)
Smoking cessation	1 out of 3 smokers	1 out of 3 smokers

^a^*n* (%) using the web site at least once during this period

Across both groups, women received mean (95% CI) 90 (88-98) % of their scheduled week 1, 4 and 8 calls and 91 (86-95%) of their scheduled e-mails. Mean (95% CI) 90 (88-98) % of scheduled calls, 91 (86-95%). There was good engagement with the self-monitoring website. Ninety-seven percent of women used the website at least once in the first 3 months, 87% 3-6 months, 83% 6-9 months and 79% 9-12 months. A number of women in the BCPP and MDPP groups were referred to NHS behaviour change services; PA referral services 8 (18%) BCPP and 23 (28.4%) MDPP, smoking cessation 1 out of 3 smokers BCPP and 5 out of 6 smokers MDPP, and alcohol services 0/8 BCPP and 4/22 (4.9%) with an AUDIT score of greater than or equal to 8 MDPP by their allocated dietitian.

### Participant satisfaction

Anonymous satisfaction questionnaires for the overall programme were completed by 71/88 (81%) of those who completed the study at 12 months (26, 57% of the BCPP group and 45, 55% of the MDPP group) (Tables 6 and 7).

**Table 6:** Anonymous participant feedback for the overall programme completed by 71/88 (81%) of those who completed the study at 12 months (26, 57% of the BCPP group and 45, 55% of the MDPP group)

Elements of the programme	Very or extremely satisfied (ranked 8/10 to 10/10)
**BCPP and MDPP groups**	
Diet advice and meal plans	53/71 (75%)
PA advice and plans	36/71 (51%)
Dietitian telephone reviews	68/71 (96%)
Dietitian weekly individualised e-mails 69/71	69/71 (97%)
Automated e-mails during 6-12 months	51/71 (72%)
**MDPP group**	
NHS Health Check assessments	44/45 (96%)
Communication of their personalised CVD and T2D risk	45/45 (100%)

**Table 7:** Website evaluation was completed by 109/126 (87%) of women at either 3 or 6 months

	Agreed or strongly agreed, *n* (%)
I think that I would like to use this website frequently.	76 (70%)
I thought the website was easy to use.	92 (84%)
I felt very confident using the website.	92 (84%)
I think that I would need the support of a technical person to be able to use this website.	3 (3%)
I found the website very cumbersome to use.	9 (9%)

Website evaluation was completed by 109/126 (87%) of women. The phone and e-mail feedback was well received. Women understood and positively evaluated the CVD and T2D risk feedback and dietary elements of the programme. However, they were less confident to follow the PA plans.

Free text feedback showed that women felt their self-efficacy to undertake PA would increase with a demonstration class or DVD. They emphasised the need for review calls to be scheduled, and that feedback e-mail should be personalised and not generic. They valued the advice from the dietitian who was considered a credible source of information. A number of changes to the website were suggested including a forum to facilitate peer-to-peer communication and support, an ‘ask the expert’ function, and adding an average weight loss line to their weight tracker so they could compare their weight loss with the average of the other participants.

### Adverse events and harms associated with the programmes

No study or intervention related serious or unexpected adverse events occurred. Completer-only analysis showed a small reductions in state anxiety score with the BCPP (*n* = 37) −0.7 (−4.6 to 3.2) and MDPP (*n* = 60) −3.5 (−6.7 to −0.4), and an increase in EQ-5D-5L scores in the BCPP (*n* = 40) 4.1 (0.6 to 7.6) and MDPP groups (*n* = 60) 7.3 (3.6 to 11.1) (Table 8). The proportion reporting a high state anxiety score (≥ 40) at baseline was 6 (16.2%) BCPP and 18 (30%) MDPP compared to 7 (18.9%) BCPP and 15 (25%) MDPP and at 6 months.

**Table 8:** Changes in weight, lifestyle behaviours and self-rated health and anxiety across the 12-month programmes

	Breast cancer prevention programme, *N* = 45			Multiple disease prevention programme, *N* = 81		
	Baseline	6 months	12 months	Baseline	6 months	12 months
Weight (kg)^a*^	82.4 (78.3-86.4)	76.7 (75.4-77.8)	77.2 (75.3-79.2)	83.7 (80.8-86.8)	76.8 (75.2-78.4)	77.0 (75.6-78.5)
≥ 5% weight loss^a**^		31 (69)	26 (58)		55 (68)	46 (57)
Body fat (kg)^a*^	33.3 (30.4-36.1)	28.6 (25.2-32.0)	29.1 (25.8-32.3)	34.4 (32.5-36.3)	29.6 (27.6-31.6 )	29.8 (27.6-31.8)
Minutes of moderate physical activity/day (IPAQ)^a*^	117 (99-136)	126 (108-143)	N/A	110 (96-124)	117 (105-130)	N/A
Alcohol intake (g/week)^a***¥^	88.2 (0-175)	25.6 (0-62)	N/A	93.0 (18-93)	29.4 (0-90)	N/A
Alcohol, AUDIT score^a***^	4 (3-7)	3 (2-5)	N/A	5 (3-8)	4 (3-7)	N/A
AUDIT score ≥ 8^a**^	8 (17.8)	4 (8.9%)	N/A	22 (27.2)	14 (17.3%)	N/A
Saturated fat (g/day)^a*¥^	28.5 (25.4-31.7)	19.0 (16.8-21.1)	N/A	25.9 (23.6-28.1)	18.4 (16.5-20.2)	N/A
Saturated fat > 20 g/day^a**^	29 (87.9%)	12 (36.4%)	N/A	40 (72.7)	19 (34.5)	N/A
State train anxiety^b*^	30.3 (25.9-34.6)	28.0 (24.1-31.9)	N/A	33.5 (29.9-37.1)	29.8 (26.4-33.3)	N/A
State trait anxiety > 40^b**^	6(16.2)	7 (18.9)	N/A	18 (30%)	15 (25%)	N/A
EQ59-VAS^b*^	79.6 (75.1-84.1)	83.7 (79.8-87.6)	N/A	78.4 (74.6-82.2)	86.0 (82.6-89.3)	N/A

^a^Baseline observation carried forward

^b^Completers only BCPP *n* = 37 MDPP *n* = 60

*Mean (95% CI), **n (%), ***Median (25th and 75th centile), *N/A* not available

^¥^Food diaries were completed by 33 (73%) of the BCPP and 55 (68%) of the MDPP groups at baseline

### Primary outcome for the planned definitive trial

Both programmes achieved good and comparable reductions in weight (Table 8). Mean (95% CI) BOCF percentage weight loss at 12 months were −7.6 (−9.8 to −5.4) % in the BCPP group and −7.4 (−9.1 to −5.7) % in the MDPP group (*p* = 0.952). BOCF weight loss at 12-months was −6.01 (−7.87 to −4.1) kg in the BCPP group and −6.2 (−7.8 to −4.71) kg in the MDPP group (*p* = 0.673), with respectively 58 and 57% and achieving weight loss of ≥ 5% (*P* = 0.915). The level of weight loss associated with reduced risk of disease [21–24].

### Changes in other self-reported health behaviours

The proportion reporting at risk drinking (AUDIT score ≥ 8) reduced from 8 (17.5%) in the BCPP and 22 (27.2%) in the MDPP at baseline to respectively 4 (8.9%) and 14 (17.3%) at 6 months. Likewise, the proportion of women reporting saturated fat intakes above the recommended intake of 20 g/day reduced from 29 (87.9%) in the BCPP and 40 (72.7%) in the MDPP at baseline to respectively 12 (36.4%) and 19 (34.5%) at 6 months. Both groups also reported modest increases in moderate intensity PA at 6 months. One woman from each group had stopped smoking by 6 months and was still not smoking at the 12-month follow-up.

## Discussion

### Main findings

The BCPP and MDPP appear feasible with good uptake, retention, fidelity, patient satisfaction and no evidence of harms. The MDPP highlighted previous unknown risk of CVD and T2D but does not appear to increase engagement with behaviour change beyond a standard BCPP amongst women attending breast screening. Both programmes showed good weight loss.

Overall uptakes to the mailed invitation to both programmes were around 10%. This is comparable to previously reported uptakes to behaviour change programmes when written invitations are given to women when they attend the NHSBSP, or when postal invitations are sent to women at increased risk of BC [38]. This feasibility study highlights potential strategies to increase recruitment to weight loss programmes within the NHSBSP. Firstly, focussing on women at high or above average BC risk who had uptakes of 14-20% compared to 4-10% for women at average or low BC risk reported herein and previously within this cohort [17]. Higher risk women will also derive a greater absolute risk reduction from changes in health behaviours compared to low risk women [39, 40].

Future programmes could include women with existing health conditions. Significant proportions of women who were interested to join the programmes were ineligible due to pre-existing weight-related health conditions (7.1% BDPP and 12.0% of the MDPP group). We presume these weight-related health conditions were not currently being addressed within existing NHS weight loss/behaviour change services. Inclusion of these women would have given uptakes of 18.1 to BCPP and 23.0% to MDPP. Lastly, recruiting women at their mammogram appointments and using remote collection of outcome measures such as weight would overcome identified barriers of time and travel around additional appointments reported by women who declined to join this study.

Both programmes appear highly effective for weight loss and behaviour change. The mean weight loss at 12 months over 6 kg using baseline carried forward imputation compares favourably with outcomes from group-based commercial (2-4 kg), internet (1.9 kg) and primary care programmes (0.7-3.6 kg) [41].

There was good engagement with the website and phone-supported programmes. This hybrid face-to-face and remotely delivered programme allowed women to receive initial individual face-to-face advice from a health care professional combined with the website that allows self-monitoring and prompt regular feedback from the health care professional. Such programmes have been found to be both more and less effective compared to standard face-to-face programmes, dependent on the level of user engagement achieved [42].

Most previous website programmes have been tested amongst younger cohorts. The few studies amongst older women concur with our findings of good engagement [43–45], and the potential for their utility amongst women of breast screening age. The 2018 Office of National Statistics bulletin reported large increases in internet usage amongst older women. In 2018, 98% of women aged 44-54 years had used the internet in the past 3 months, compared to 92% of 55-64 year olds and 80% of 65-74 year olds [46].

There has been minimal research on the feasibility or efficacy of multiple disease prevention programmes. It was thought that multiple disease risk information could be personally relevant to a larger group of women than just providing risk information on a single disease such as BC, increasing the likelihood of having a positive effect on behaviours [47]. Alternatively, it was possible multiple disease risk information could reduce motivation and self-efficacy and threaten self-integrity to change health behaviours in some individuals. We found no difference in engagement and behaviour change with a supervised MDPP compared to the BCPP.

There was no evidence of harm with the MDPP such as increased anxiety, or lower self-rated health assessed with the EQ-5D as a result of being provided with personalised risk of CVD and T2D. This is an important finding which is in line with other studies of providing risk information, despite concerns about adverse effects [48, 49]. The higher drop out in women at higher baseline risk of CVD in the MDPP group may be an incidental finding. CVD risk information by itself has minimal effect on health behaviours [50]. In the recent INFORM trial, neither genotypic nor phenotypic coronary heart disease risk information influenced the efficacy of a web-based lifestyle programme for changes in PA, diet or weight [51]. Future studies should assess whether CVD risk information impacts on engagement and the efficacy of weight loss programmes.

### Strengths and limitations

This is one of the few studies to test the feasibility and acceptability of a BC prevention and multiple disease prevention programmes in women in the breast screening programme. Also, one of the few to test website and phone programmes amongst middle-aged women. Twelve-month data including 6 months of website-only support provides an indication of the long-term effects of the programmes for weight loss maintenance.

Key limitations that a future effectiveness trial should address are as follows. The study invited a selected group of women who had previously been recruited to the PROCAS study, who were recruited some time after their attendance at breast screening. Studies need to test uptake to the programmes in an unselected cohort at the time of breast screening. In addition, all trial procedures were conducted at a research unit. The future trial should ideally be in a screening site or a nearby community location to inform how these programmes could be integrated within the NHSBSP or be fully remote or online. It should also include a number of centres, and not just one as tested in this feasibility study.

The 68% retention at 12 month compares favourably with weight loss studies which often have retention rates of below 50%. However, our attrition rate of 32% is above the recommended level of 20% and so raises concerns about attrition bias and the validity of the study [52]. Future studies would be conducted with a clinical trials unit where enhanced; administrative support could help reduce attrition. Further strategies to reduce attrition bias could include financial incentives and additional contact with participants in the weight loss maintenance phase of the intervention [53]. We did not include a control group, so we do not know the likely uptake and retention in a control group, and how best they could be kept engaged.

Secondary trial end-points (e.g. weight) were assessed by members of the research team which sometimes included the research dietitians delivering the intervention who were not blinded to the intervention. The study included two intervention groups and did not include a no intervention control group which would be required in a definitive randomised trial.

Further participant involvement and engagement (PPIE) work can help refine the invitation and recruitment strategy to the programmes. This could include a personalised letter of endorsement of the trial from their family doctor [54]. PPIE work is specifically required to promote uptake amongst women from black, Asian, minority ethnic and socially deprived groups who had low uptakes to this study, as seen with previous breast screening and cancer trials [55]. Other potential strategies, such as face-to-face introduction to the trial at the time of screening and telephone follow-up after the mailed invite [56] are unlikely to be feasible due to the size of the target population.

### Implications and future research

We assessed the feasibility and acceptability of a BCPP or MDPP amongst women in the breast screening programme and whether there were any additional benefits with the MDPP programme, to determine which programme was worthy of further investigation in a large scale RCT within the NHSBSP. Both programmes met the NICE criteria for the efficacy of weight loss programmes, i.e. at least 60% of participants are likely to complete, average weight loss is at least 3%, and at least 30% of participants lose ≥ 5% of their initial weight [57]. The simpler, lower cost BCPP would be the preferred test programme to assess long-term behaviour change and weight loss and reduced risk of BC vs. standard care and other weight- and health-behaviour-related conditions.

Increased coverage and appropriate medication are current targets for the NHS Health Check programme [13]. Given this, future trials could assess the impact of offering a MDPP around the time of breast screening to increase coverage of CVD and T2D risk assessment and whether a one-stop shop can increase NHS efficiency and potential uptake to the NHSBSP.

Dietitians delivered the test programmes. Future programmes could consider provision of original dietary advice by a dietitian as a trusted source of knowledge, with ongoing support from a health trainer to reduce costs. This model of delivery has been favourably evaluated in other weight loss settings [58] and likely to be more effective than programmes which are entirely delivered by non-specialists which have achieved only modest weight loss [59]. Further work is required to improve the PA component of the programme which had modest effects on PA.

## Conclusion

The MDPP identified previously unknown CVD and T2D risk factors but does not appear to increase engagement with behaviour change beyond a standard BCPP amongst women attending breast screening. The results suggest a definitive effectiveness trial of the BCPP intervention is warranted. Weight loss achieved with a BCPP will reduce risk of BC as well as CVD and T2D and other weight-related cancers and health conditions.

## Supplementary Information

**Additional file 1: Supplementary Table 1.** Multivariable logistic regression of the association between baseline risk of type 2 diabetes and cardiovascular disease and withdrawal and percentage weight loss within the multiple disease prevention group (*n* = 81). Adjusting for age, Townsend deprivation vation, smoking status, BMI and breast cancer risk category

**Additional file 2: Supplementary Table 2.** Completeness of weight and self -reported lifestyle, anxiety and health status endpoints

## Data Availability

The trial protocol and all datasets used and analysed during the current study are available from the corresponding author on reasonable request.

## Abbreviations

BC: Breast cancer
CVD: Cardiovascular disease
T2D: Type 2 diabetes
NHSBSP: NHS Breast Screening Programme
BMI: Body mass index
BOCF: Baseline observation carried forward
PROCAS: Predicting of Cancer at Screening
BCPP: Breast cancer prevention programme
MDPP: Multiple disease prevention programme
