# Supplementary material for: Testing a breast cancer prevention and a multiple disease prevention weight loss programme amongst women within the UK NHS breast screening programme—a randomised feasibility study
Source: Pilot Feasibility Stud. 2021 Dec 20;7:220. doi: 10.1186/s40814-021-00947-4 (PMC8690875; doi:10.1186/s40814-021-00947-4)
Supplement: Supplementary file 1 — Additional file 1: Supplementary Table 1. Multivariable logistic regression of the association between baseline risk of type 2 diabetes and cardiovascular disease and withdrawal and percentage weight loss within the multiple disease prevention group (n = 81). Adjusting for age, Townsend deprivation vation, smoking status, BMI and breast cancer risk category [file 40814_2021_947_MOESM1_ESM.docx]

Supplementary Table 1 Multivariable logistic regression of the association between baseline risk of type 2 diabetes and cardiovascular disease and withdrawal and percentage weight loss within the multiple disease prevention group (n = 81)

|  | **Withdrawal from the study** | | | |
| --- | --- | --- | --- | --- |
|  | **Unadjusted** |  | **Adjusted*** |  |
|  | **OR (95%CI)** | **P value** | **OR (95%CI)** | **P value** |
| Diabetes: QDiabetes | 1.05 (1-1.1) | 0.05 | 1.03 (0.94,1.11) | 0.53 |
| First degree relative with Diabetes: No vs Yes | 1.46 (0.52-4.04) | 0.47 | 1.25 (0.33,4.71) | 0.74 |
| CVD: QRISK2 | 1.08 (0.97-1.2) | 0.17 | 1.36 (1.08,1.8) | 0.02 |
| First degree relative with CVD: No vs Yes | 2.26 (0.81-7.03) | 0.13 | 1.62 (0.44,6.47) | 0.47 |
| CVD risk: Heart age vs actual age | 1.19 (1.07-1.35) | 0.003 | 1.22 (1.03,1.46) | 0.02 |
|  |  |  |  |  |

Adjusting for age, Townsend deprivation vation, smoking status, BMI and breast cancer risk category
