# Supplementary material for: Testing a breast cancer prevention and a multiple disease prevention weight loss programme amongst women within the UK NHS breast screening programme—a randomised feasibility study
Source: Pilot Feasibility Stud. 2021 Dec 20;7:220. doi: 10.1186/s40814-021-00947-4 (PMC8690875; doi:10.1186/s40814-021-00947-4)
Supplement: Supplementary file 2 — Additional file 2: Supplementary Table 2. Completeness of weight and self -reported lifestyle, anxiety and health status endpoints [file 40814_2021_947_MOESM2_ESM.docx]

Supplementary table 2 Completeness of weight and self -reported lifestyle, anxiety and health status endpoints

|  | Breast cancer prevention programme  N = 45 n (%) | | | | Multiple disease prevention programme  N = 81 n (%) | | | |
| --- | --- | --- | --- | --- | --- | --- | --- | --- |
|  | Baseline | 3 month | 6 months | 12 months | Baseline | 3 month | 6 months | 12 months |
| Retention to the study | 45  100% | 42  93% | 41  91% | 33  73% | 81  100% | 73  90% | 65  80% | 53  65% |
| Weight | 45  100% | 42  93% | 41  91% | 33  73% | 81  100% | 73  90% | 65  80% | 53  65% |
| Body fat  (bioelectrical impedance) | 45  100% | 42  93% | 41  91% | 33  73% | 81  100% | 73  90% | 65  80% | 53  65% |
| Physical activity / day (IPAQ) ^a*^ | 43  96% | 42  93% | 37  82% | Not collected | 79  98% | 68  84% | 57  70% | Not collected |
| Alcohol  Audit questionnaire | 45  100% | 42  93% | 39  87% | Not collected | 81  100% | 72  89% | 65  80% | Not assessed |
| Saturated fat  7 day food diary | 42  93% | 32  71% | 29  64% | Not collected | 81  100% | 65  80% | 58  71% | Not assessed |
| Self- reported smoking status | 45  100% | 42  93% | 41  91% | Not collected | 81  100% | 75  93% | 70  86% | Not assessed |
| State trait anxiety ^b*^ | 45  100% | 39  87% | 37  82% | Not collected | 78  96% | 71  88% | 62  77% | Not assessed |
| EQ59 health status | 45  100% | 41  91% | 40  89% | N/A | 81  100% | 70  86% | 63  78% | Not assessed |
